# Sandblasted and Acid Etched Titanium Dental Implant Surfaces Systematic Review and Confocal Microscopy Evaluation

**DOI:** 10.3390/ma12111763

**Published:** 2019-05-30

**Authors:** Gabriele Cervino, Luca Fiorillo, Gaetano Iannello, Dario Santonocito, Giacomo Risitano, Marco Cicciù

**Affiliations:** 1 Department of Biomedical and Dental Sciences and Morphological and Functional Imaging, Messina University, 98122 Messina ME, Italy; gcervino@unime.it (G.C.); lfiorillo@unime.it (L.F.); gaet.doc@gmail.com (G.I.); 2 Multidisciplinary Department of Medical-Surgical and Odontostomatological Specialties, University of Campania “Luigi Vanvitelli”, 80100 Naples NA, Italy; 3 Department of Engineering, Messina University, 98122 Messina ME, Italy; dsantonocito@unime.it (D.S.); grisitano@unime.it (G.R.)

**Keywords:** dental implants, surface properties, osseointegration, bone-implant interface

## Abstract

The field of dental implantology has made progress in recent years, allowing safer and predictable oral rehabilitations. Surely the rehabilitation times have also been reduced, thanks to the advent of the new implant surfaces, which favour the osseointegration phases and allow the clinician to rehabilitate their patients earlier. To carry out this study, a search was conducted in the Pubmed, Embase and Elsevier databases; the articles initially obtained according to the keywords used numbered 283, and then subsequently reduced to 10 once the inclusion and exclusion criteria were applied. The review that has been carried out on this type of surface allows us to fully understand the features and above all to evaluate all the advantages or not related. The study materials also are supported by a manufacturing company, which provided all the indications regarding surface treatment and confocal microscopy scans. In conclusion, we can say that, thanks to these new surfaces, it has been possible to shorten the time necessary to obtain osseointegration and, therefore, secondary stability on the part of implants. The surfaces, therefore, guarantee an improved cellular adhesion and thanks to the excellent wettability all the biological processes that derive from it, such as increases in the exposed implant surface, resulting in an increase in bone-implant contact (BIC).

## 1. Introduction

### 1.1. Rationale

Currently, implants are almost all made of titanium. The most used are the endosseous screw-type, in most cases left submerged under the gingiva for a reasonable period depending on the site. Dental implantology is, therefore, subdivided into endosseous and juxta-osseous, the latter using only non-submerged fixed-grid systems and, therefore, for non-osseointegrable seat and loading modalities, if made of chrome-cobalt-molybdenum, or even osseointegratable if made in titanium and inserted with special surgical techniques favouring the new bone formation above their structure.

The endosseous implantology is currently the most widespread, and uses cylindrical/conical implants (the actual implant body) threaded on the outside and with an internal connection of varying conformation for the emerging part (stump) and rarely cylinders or cones without external threads, but with similar internal connection systems for the abutment, screws full of a single body (implant body and abutment made from solid and, therefore, without any connection), blades and needles. According to the surgical protocol we will, therefore, have submerged and non-submerged implantology (transmucosal); based on the timing of use (functionalization) we will have immediate, anticipated, deferred loading. The most used material for the production of implants is titanium, in a commercially pure form or in its alloys for dental use, a biocompatible material that does not involve reactions from the organism (popularly, but erroneously, known as rejection). The implants, positioned in the patient’s bone will be strongly incorporated into it by the physiological mechanisms of bone regeneration, i.e., osteointegration will take place both in the event of delayed loading and in the case of immediate loading. The trend of recent years has been to accelerate dental rehabilitation as much as possible, also undertaking immediate rehabilitation protocols. This has been possible thanks to the improvement of the geometries of the dental implants, but also to the improvement of the surfaces, thanks to surface treatments that allow a better bone-implant interaction. We have evaluated these surfaces and we will analyse them in detail in the following sections, once we have also made a quick reference to titanium surface treatments.

### 1.2. Objectives

The objective of this study is to evaluate the characteristics of sandblasted and acid etched (SA) implant surfaces, their treatment methods and surface interactions [1,2,3,4,5,6,7,8,9,10]. The authors then carry out a systematic review of the results in the literature on the study, the characteristics of this surface. Furthermore, to support the literature, a confocal microscopic analysis of a surface SA [11,12,13,14,15,16]. Implantology (dental) means that set of surgical techniques designed to functionally rehabilitate a patient suffering from total or partial edentulism through the use of dental implants or devices, metallic or not, surgically inserted into the mandibular or maxillary bone, or above it but under the gingiva, acting in turn to allow the connection of fixed or mobile prostheses, for the restitution of the masticatory function. These implants can be of different shapes, inserted in different locations with different techniques and then connected to the prosthesis at different times.

## 2. Material and Methods

### 2.1. Protocol and Registration

This review is registered at PROSPERO with ID number 136038. PROSPERO is an international database of prospectively registered systematic reviews in health and social care.

### 2.2. Eligibility Criteria

The following focus question was developed according to the population, intervention, comparison, and outcome (PICO) study design:

- What are the surface characteristics of sandblasted and acid etched dental implants?

### 2.3. Information Sources

The search strategy incorporated examinations of electronic databases, supplemented by hand searches. A search of PubMed, Dentistry, and Oral Sciences Source, for relevant studies published in the English language. A hand search of the reference lists in the articles retrieved was carried out to source additional relevant publications and to improve the sensitivity of the search.

### 2.4. Search

The keywords used in the search of the selected electronic databases included the following:

- (“sandblasted” OR “acid etched”) AND (“dental implant” OR “implantology”)

The choice of keywords was intended to collect and to record as much relevant data as possible without relying on electronic means alone to refine the search results.

### 2.5. Selection of Studies

Two independent reviewers singularly analysed the obtained papers in order to select inclusion and exclusion criteria. For the stage of reviewing full-text articles, a complete independent dual revision was performed.

### 2.6. Study Selection

After the first literature analysis, all article titles were screened to exclude irrelevant publications, case reports and non-English language publications. Then, studies were not selected based on data obtained from screening the abstracts. The final stage of screening involved reading the full texts to confirm each study’s eligibility, based on the inclusion and exclusion criteria. 

The full text of all studies of possible relevance was obtained for assessment against the following inclusion criteria:

- All randomized clinical trials about the use of SA implant surfaces on humans;
- Clinical follow up about SA implant surface use on humans; and
- All confocal studies on SA surfaces.

The applied exclusion criteria for studies were as follows:

- Studies involving patients with other specific diseases, immunologic disorders, or other oral risk-related systemic conditions;
- Not enough information regarding the selected topic; or
- No access to the title and abstract in the English language.

The review included studies on humans and animal published in the English language. Letters, editorials, and PhD theses were excluded. The review included all human prospective and retrospective follow-up studies and clinical trials, cohort studies, case–control studies, and case series studies, animal studies and literature review published, on sandblasted and acid etched dental implants uses for rehabilitation and implantology. The data were independently extracted from studies in the form of variables, according to the aims and themes of the present review, as listed onwards.

### 2.7. Data Collection Process

Data were collected from the included articles and arranged in the following fields (Table 1):

Author (Year)—Authors and year of publication;
Sample Size—Size of sample evaluated;
Torque—Implant positioning torque nm (Newton/meter);
Follow up—Implant follow up period (maximum value);
Statistic—Statistical results; and
Type of Parameters evaluated—Evaluated parameters about the implant.

### 2.8. Data Items

PICO has been used to conduct the review, and of any assumptions or simplifications made.

### 2.9. Risk of Bias Assessment

Assessment of risk of bias was undertaken by two authors during data extraction process (G.C. and L.F.). For the included studies, this was conducted using the Cochrane Collaboration’s two-part tool for assessing risk of bias [17,18,19,20,21,22]. An overall risk of bias was then assigned to each trial according to Higgins et al. [19]. The levels of bias were classified as follows: low risk, if all the criteria were met; moderate risk, when only one criterion was missing; high risk, if two or more criteria were missing; and unclear risk, if too few details were make a judgement of certain risk assessment.

### 2.10. Implantology and Different Surfaces

A dental implant is an alloplastic structure inserted by means of appropriate surgery, in the structure of the jaw bones to rehabilitate a patient who has a condition of edentulism. The implants have characteristics related to the materials with which they are made and to their geometry, macroscopic and microscopic. The material normally used is commercially pure titanium, this material has characteristics of biocompatibility, low density, electrochemical stability, high mechanical strength and sufficient rigidity [23,24,25,26,27,28,29]. The macroscopic implant geometries evaluate macroscopic differences in the shape of dental implants. In this case the turns of the dental implant with different dimensions, number and pitch may be affected. Coils may be present or not [30,31,32,33,34,35,36,37,38,39,40]. The function of the loops is to efficiently distribute the masticatory load and to guarantee stability in the early post-surgical phases of the implant [41,42,43]. On the other hand, secondary or surface geometries are the basis of the osseointegration potential of the implant. The methods adopted for surface treatment are different. We certainly distinguish additive and subtractive techniques. Subtractive techniques include:

- Sandblasting: the bombardment of titanium surfaces with granules of variable diameter of oxides (titanium dioxide, aluminum oxide, zirconium dioxide and silicon carbide)
- Acid etching: carried out with sulfuric, hydrofluoric or hydrochloric acid according to different protocols.
- Combination of sandblasting and acid etching (SA)
- Oxidation in a galvanic bath
- Elettroerosion (EDM)

**Figure 1:**
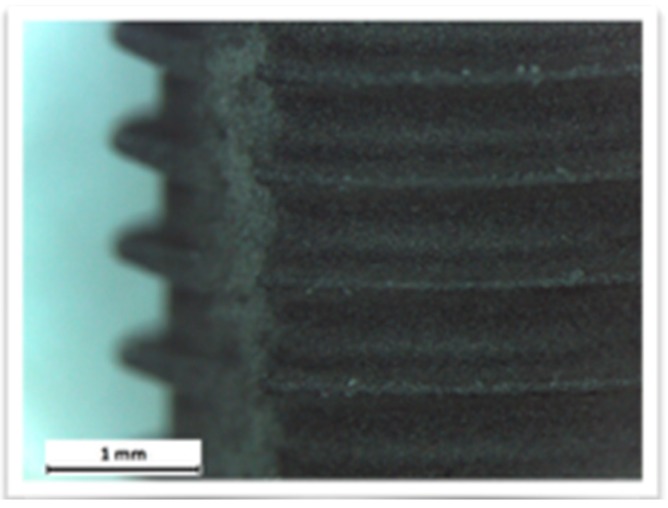
Competitor 1.

**Figure 2:**
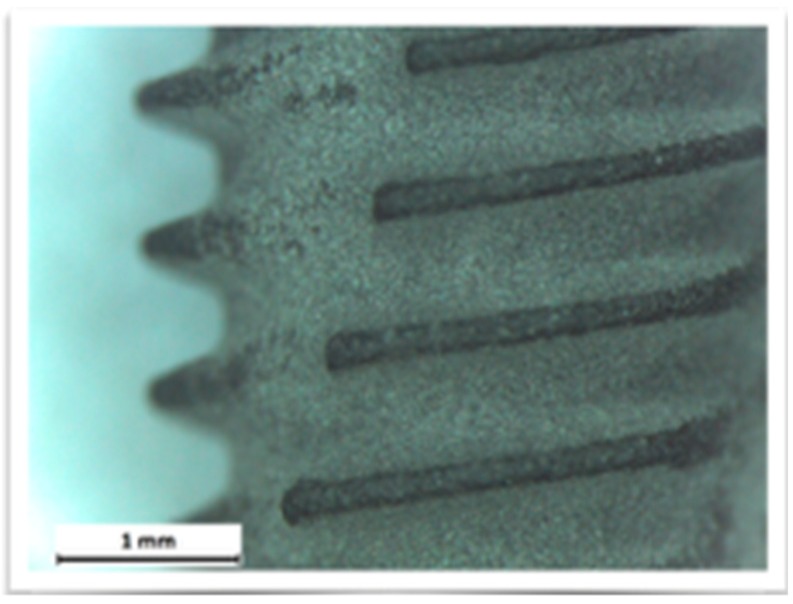
Test group surface.

**Figure 3:**
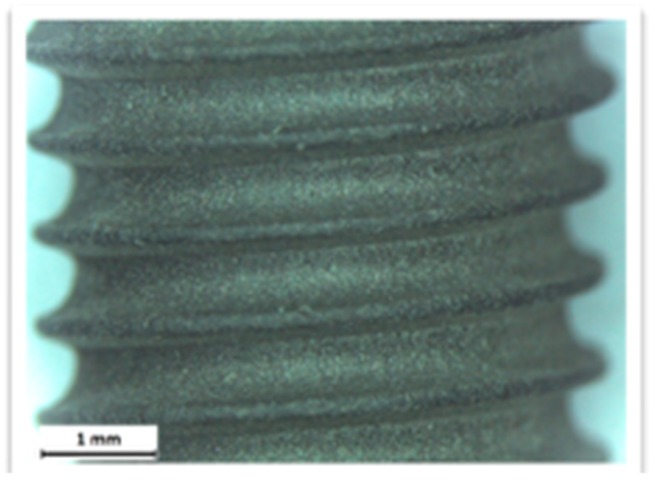
Competitor 2.

The additive techniques use a flow of plasma to deposit on the implant surface different materials, such as titanium powder, hydroxyapatite powder, or titanium microspheres. The degree of bacterial proliferation on these surfaces is also influenced by the type of surface [44,45,46,47,48,49,50,51,52] (Figure 1, Figure 2 and Figure 3).

### 2.11. 3D Confocal Microscopy

The confocal microscope is an optical microscope, a scientific instrument that is based on a technology aimed at significantly increasing the spatial resolution of the sample, eliminating the halos due to the light diffused by the out-of-focus planes of the preparation.

**Figure 4:**
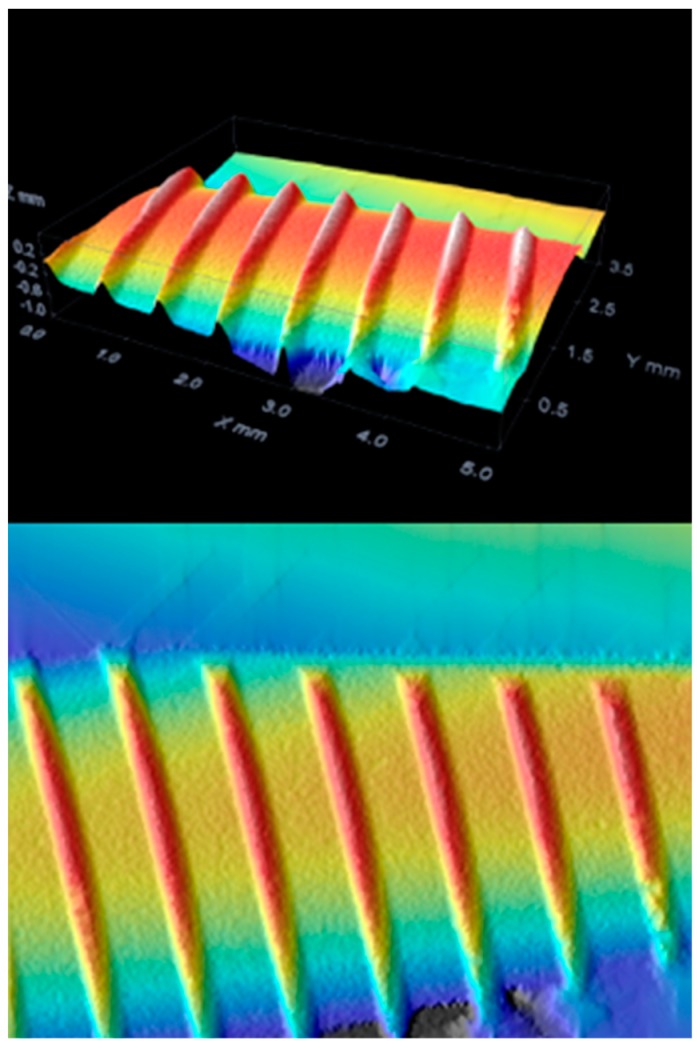
Competitor 1 confocal microscopy.

**Figure 5:**
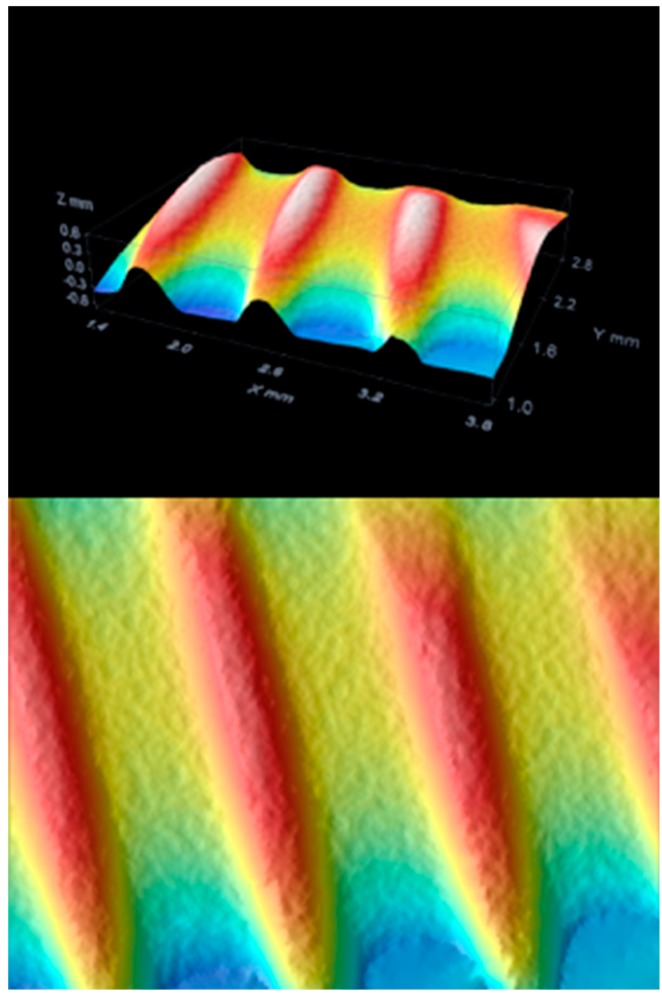
Test group confocal microscopy.

**Figure 6:**
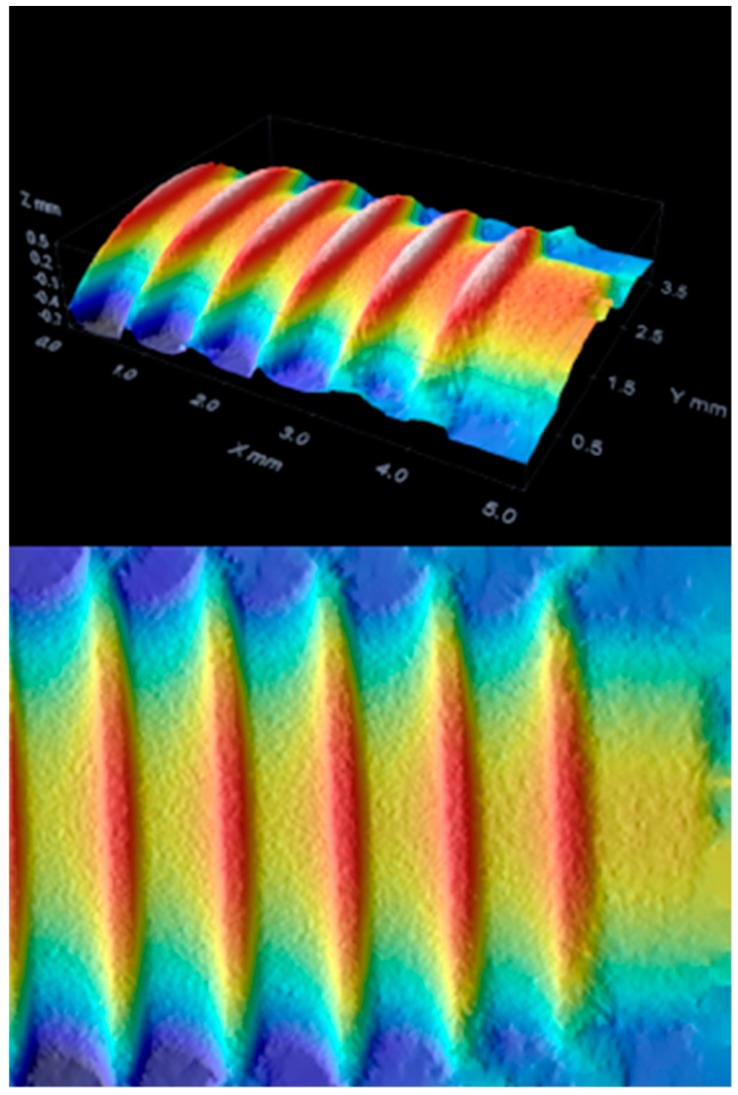
Competitor 2 confocal microscopy.

**Figure 7:**
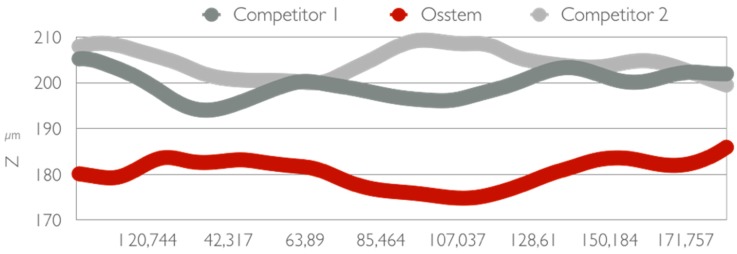
Roughness differences.

The instrument operates in the conventional field of the magnifications of normal optical microscopy, and is schematically constituted by a normal transmission microscope to which an apparatus that deals with illuminating and detecting the image of an illuminated sample with a point-to-point scan is superimposed. There are different techniques to achieve this: rotating disk (Nipkow disk), programmable array microscopes (PAM), and laser. The latter type, the most widespread and called CLSM (confocal laser scanning microscope), is an advanced fluorescence microscope that allows to focus with extreme precision a laser on the preparation, greatly increasing the resolution and depth of field. Its light source consists of one or more lasers, generally semiconductor, for each different frequency of excitation required. The light beam direction mechanism is managed by computerized systems. The images obtained, by synchronizing the detection device with the excitation beam, are particularly defined and spectacular, and can allow the different molecules present in the preparation to be highlighted in different colors, making it possible to appreciate their three-dimensionality [53,54] (Figure 4, Figure 5, Figure 6 and Figure 7).

### 2.12. Risk of Bias across Studies

There were several limitations present in the current review. The current review includes studies written in English only, which could introduce a publication bias. There were various degrees of heterogeneity in each study design, case selection, and treatment provided among studies.

## 3. Results

The results were collected from all the articles taken into consideration, articles that discuss dental materials and their use in the field of rehabilitative and restorative dentistry. In this article we have taken into consideration therapeutic planning, aesthetic and functional rehabilitation articles, as well.

### 3.1. Inclusion Study Flow

**Figure 8:**
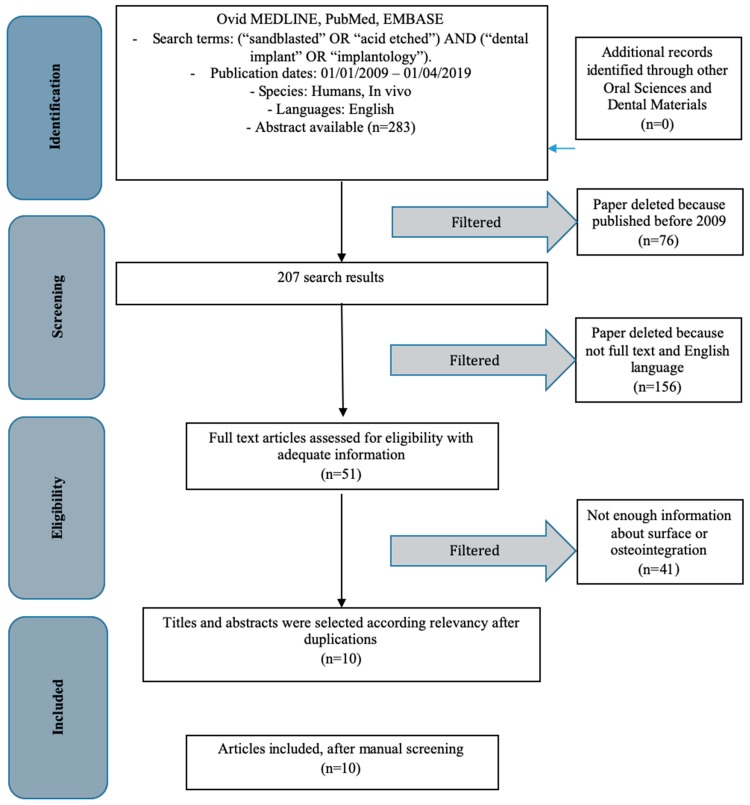
PRISMA flow chart.

Article review and data extraction were performed according to PRISMA flow diagram (Figure 8). The initial electronic and hand search retrieved 283 articles. After titles and abstracts were reviewed, only 10 articles were included. 

### 3.2. Study Characteristics and Summary Measures

During the selection of the studies the individual characteristics of each one were evaluated. The characteristics assessed mainly concern the clinical signs and follow up period as described in Section 2.12 (Table 2)

- Bone to implant contact (BIC)
- Bone Density
- Implant or prosthetic failures
- Soft tissue signs
- Histological or histomorphological evaluation
- RFA
- Region of interest (ROI) percentage

### 3.3. Risk of Bias within Studies

Many studies have been evaluated with an unclear risk of bias, as there is not enough information to establish the risk. Only one study presents assessments that allows a risk of low bias [55].

### 3.4. Results of Individual Studies and Synthesis of Results

The results of the individual studies were collected accordingly to the data obtained after the research as follows in Table 1.

### 3.5. Additional Analysis

In addition to reviewing and supporting the literature, a study was conducted on the surface of a test dental implants. The surface was evaluated from different points of view, first with optical microscopy and subsequently with confocal microscopy to evaluate those that are the characteristics related to surface roughness. The tested fixture has a macroscopic geometry, for details see Section 2.10 with loops at intervals of 0.8 mm and a depth of 0.25 mm. The geometry of the loops is progressive, and reach a depth of 0.5 mm in the apical direction. The surface is an SA surface. Three different implant fixtures were evaluated: A test dental implant and two other competitors, named as Competitor 1 and Competitor 2.

The images obtained with optical microscopy are shown in Figure 1, Figure 2 and Figure 3.

On examination with confocal optical microscopy, the differences between the test surface and the competitor implants are evident, as shown in the graph.

Optical microscopy does not show statistical differences between implants using surface analysis software, while confocal microscopy analysis shows a clear difference in surface roughness. This characteristic is closely linked to cell proliferation, healing of the fixture in the bone, then osseointegration and BIC [65].

## 4. Discussion

### 4.1. Summary of Evidence

There are many studies about SA surface implants and, more specifically, about tested surface implants. These articles take into consideration different topics related to implant surgery and can be summarized as follows:

The SA surfaces are, therefore, used for the construction of dental implants. After this surface treatment the implants have been subjected to strict quality controls and studies that evaluate their reliability. The studies present in the literature, as shown in Table 2, are numerous and all go to evaluate different aspects: First of all, the implant survival, hence, the clinical conditions of the implant subjected to function; the loading protocols of the dental implant, where many studies concern the prosthesis itself; the differences between screwed or cemented prostheses on implants, use of bars, built with different materials and made with different methods, and the very important studies carried out with the Finite Element Method (FEM) [93,100,101,102]; articles concerning pre-implant surgical techniques, including bone grafts, maxillary sinus augmentations or bone regeneration [103,104,105,106,107,108,109,110,111]; and, finally, the study of the bacterial flora on the surface of the dental implants. With attention we will evaluate these last works. There is talk of success of a dental implant when it meets a series of criteria once it is placed in the oral cavity and prosthesized. These criteria are related to certain clinical parameters, to the symptoms and to the functionality of implant-prosthetic rehabilitation [66,67].

According to Novellino et al. [56] implants with hydrophilic sandblasted and acid etched (SAE) surfaces osseointegrate faster than implants with SAE surfaces. SA surfaces and SAE surfaces have only a different nomenclature, but both regard sandblasted acid etched surfaces. In this study, Novellino et al. used a modified SAE surface (Acqua Neodent^®^, Crawley, West Sussex, UK) with improved hydrophilic properties. The titanium oxide layer on the surface of the implants is generally electronegative. The consequence of this particular characteristic is the reduction of the contact between the implant surface and the blood, this also being electronegative. Implants with a hydrophilic surface are characterized by a layer of electropositive titanium oxide. The physical-chemical activation of the water surface modifies the negatively-charged surface in positive, attracting ions from the blood, improving the contact as proven by in vitro studies [112]. The stability gain is faster according to radiofrequency analysis (RFA) measurement. According to Mangano et al.’s study [57] on BioMed Research International, their histomorphometric study shows the healing phases of a calcium-incorporated surface implant and an SA surface implant. According to this RCT the first type of surface increased the peri-implant endosseous healing properties. According to Schmitt et al. [55] surfaces with a biomimetic layer can improve the healing process. This study revealed a long-term effect to the biofunctionalization with a peri-implant bone formation in regions of poor bone quantity. According to Cannizzaro et al. [58] in an up to six-month loading machined and roughened flapless-placed implant, similar results have been provided. An article by Schwarz et al. in 2013 [59] says that a modified SA surface may have the potential to enhance soft tissue adhesion. Corvino et al. [60] underline that implant surface topography entails on cells proliferation. According to Karabuda et al. [61] modified sandblasted and acid etched surface demonstrated a better stability and reduced healing time, having a positive success and survival rate at the end of a 15-month follow up. D’Avila et al. [62] demonstrated how SA surfaces present better results than the machined surfaces. They evaluated BIC, and reactive oxygen species concentration on smokers [113]. According to Shibli et al. [63] bioceramic molecular impregnated surfaces heal positively. According to Khang et al. [64] SA surfaces show better success rates in the conditions of poor quality or soft bone. 

The articles taken into consideration often present different surgical techniques for the positioning of dental implants. These techniques may first of all take into account the clinical conditions of the patients, which may represent problems related to healing or, in some cases, even to the risk of the patients [114,115,116,117]. It is very difficult to manage patients with congenital bleeding disorders that require careful attention. It is necessary to emphasize the perioperative management of the patient with bleeding disorders [118]. Cases of rare bleeding disorders, such as congenital afibrinogenemia, have been reported in the literature. The bleeding manifestations with gingival bleeding were repeated in this patient. An individual approach is needed if the patient needs maxilofacial surgery [119]. Venous thromboembolism is the second most common medical complication, the second most common cause of increased length of hospital stay, the third most common cause of mortality and a significant increase in financial cost [117,120,121]. Surgical risk may also be related to local factors related to the patient, such as the presence of anatomical abnormalities, oral lesions or oral neoformations [122]. Certainly, these situations can make the placement of an implant difficult or secondary to other surgeries that may also be reconstructive [103]. Implant surgery should certainly be avoided in the case of biomolecular markers positive for potentially malignant lesions or the presence of acute or chronic inflammatory processes in progress [113,123,124]. Furthermore, before talking about the surgical phase, it is necessary to set patients with a correct pharmacological protocol and correct antibiotic prophylaxis [125,126,127]. Implant surgery, therefore, requires, after a correct anaesthesia, a mucous incision in order to set up and elevate a surgical flap, so as to highlight the underlying bone tissue and proceed with the implant preparation. This phase is not necessary in flapless techniques, which do not include incisions and flaps. Implant preparation can be done with rotating burs, osteotomes or piezo surgical instruments [128,129]. Once the preparation is complete, implant placement is performed, followed by suturing. Returning to the bioengineering discourse, rather than the surgical phase, the SA surfaces show excellent osseointegration results in agreement with all the examined works, and some works show results with modern, modified or improved surfaces. During the writing of this study it was certainly necessary to pay attention also to other implant surfaces, the trend of recent years, in fact, is to improve the surface technologies of the plants so as to guarantee better performance, performances that sometimes test the biological principles of the organism [123]. The micro- and nanostructured surfaces that have been made available on the market today [10], and the SA surfaces are excellent compromises, and thanks to the presence of long-term follow-up the literature reassures us about this. Improved dental materials technologies, and efficient and conservative surgical techniques have enabled us to rehabilitate patients in an increasingly predictable manner. Dental care tends to be more conservative than in the past, above all, thanks to advances in medicine [10,93,103,113,114,117,124,130,131,132,133,134,135].

### 4.2. Limitations

Efforts were concentrated in the study of the SA surface, although the other surfaces have been evaluated, there are excellent studies that express favourable opinions on nanotexturized surfaces that are not the subject of this review. SA surfaces, however, show excellent clinical results.

## 5. Conclusions

In conclusion, we can say that the SA surfaces obtain good results highlighted by the works both from a clinical, biomechanical and histological point of view. The healing phases are stimulated by this type of implant surface, therefore, sandblasting and acid etching appears to be a safe method that produces reliable and predictable surfaces to what emerges from the results. Furthermore, the experimental study carried out by the University of Messina provided important information regarding the SA surface of dental implants. The roughness of this implant has emerged to be greater than that of competitors, guaranteeing a whole series of clinical aspects already dealt with in the manuscript. The literature also offers good news regarding the interaction by eukaryotic and prokaryotic cells with this surface, showing also excellent characteristics in case of contamination by a biofilm and, therefore, in the progress of the peri-implant pathology. Implants built with these characteristics are reliable in accordance with the results and have good integration with hard and soft tissues.

## Figures and Tables

**Table 1:** Selected studies evaluated parameters. (✔ = histologic examination done).

Author (Year)	Sample Size	Torque	Follow up	Statistic	Type of Parameters Evaluated					
					RFA Evaluation	Histologic	Histomophometric	Prothestic Failures	Implant Failures	In Vitro
Novellino et al. (2017) [56]	21	35.125 ± 4.498	1 y	*p* < 0.01	CG: 42–81 SA					
					TG: 32.5–82.5 MSA					
Mangano et al. (2017) [57]	10			Not significant		✔	TG: BIC 35.9% BD 31.8			
							CG: BIC 29.9% BD 32.5%			
Schmitt et al. (2015) [55]	10			BIC 0.002;		✔	Machined			
							SA			
							Hydroxyapatite surface			
Cannizzaro et al. (2016) [58]	50	>50	6 m	Not significant				✔	✔	
Schwarz et al. (2013) [59]	30		8 w	*p* < 0.05		✔				
Corvino et al. (2012) [60]	15		2 m	BIC *p* = 0.028		✔	✔			✔
Karabuda et al. (2010) [61]	22	CG: 25.48	6 w	*p* < 0.05	CG: 58.21			✔	✔	
		TG: 23.75			TG: 58.15					
D’Avila et al. (2010) [62]	7		2 m	BIC significant			Machined: BIC 10.40%		✔	
							Sandblasted: BIC 22.19%			
Shibli et al. (2010) [63]	10		2 m	BIC *p* < 0.05;BA not significant		✔	✔		✔	
Khang et al. (2001) [64]	97		6 m					✔	✔	

**Table 2:** Topics of SA surface studies.

Osstem SA^®^ (Seoul, South Korea) Surface Field of Study	References
Implant survival rate	[66,67]
Implant surface	[11,12,13,14,15,16]
Implant loading time	[68,69]
Prosthetic study	[70,71,72,73,74,75,76,77,78]
Maxillary sinus lifts and implant	[79,80,81,82]
Bone augmentation and implant	[83,84,85,86,87]
Implant stability	[88,89,90,91,92]
FEM on implant components	[93]
Microbial flora on implant	[94]
Implant studies on animals	[95,96,97,98,99]

## Abbreviations

BIC	Bone to implant contact;
BD	Bone area;
ROI	Reactive oxygen species;
CG	Control group;
TG	Test group;
SA	Sandblasted acid etched surface;
MSA	Modified SA;
Ti	Titanium;
EDM	Electroerosion;
RFA	Radiofrequency analysis;
FEM	Finite Element Method.
